# An interpretable machine learning model predicts frailty risk in middle-aged and older adults with gastrointestinal disease: a longitudinal study

**DOI:** 10.1038/s41598-026-50348-x

**Published:** 2026-04-28

**Authors:** Yin Chen, Mingyu Chen

**Affiliations:** 1 Department of General Surgery, The Affiliated Xuancheng Hospital of Wannan Medical University (Xuancheng People’s Hospital), Xuancheng, 242000 P. R. China; 2 Department of Internal Medicine, Guangwai Hospital (Guangwai Geriatric Hospital) of Xicheng District, No. 2A, Sanyili, Xicheng District, Beijing, 100053 P. R. China

**Keywords:** Frailty, Machine learning, CHARLS, Shapley additive explanation, Gastrointestinal disease, Computational biology and bioinformatics, Diseases, Health care, Medical research, Risk factors

## Abstract

Frailty is a significant health concern in the aging global population, particularly among middle-aged and older adults with gastrointestinal disease (GID). Early detection of individuals at increased risk is critical for implementing timely preventive and therapeutic interventions. This study aimed to develop and validate an interpretable machine learning (ML) model to assess frailty risk in this population. To overcome the “black box” nature of conventional ML models, we integrated Shapley Additive exPlanations (SHAP), which helps identify key predictors of frailty and improve the interpretability of the model’s decision-making process. This study analyzed data from the 2013-2015 survey waves of the China Health and Retirement Longitudinal Study (CHARLS). To identify the most predictive variables for frailty, we employed a dual-method approach combining the Boruta algorithm and Least Absolute Shrinkage and Selection Operator (LASSO) regression. We applied ten different ML algorithms to develop prediction models: Logistic Regression (LR), Support Vector Machine (SVM), Random Forest (RF), Gradient Boosting Classifier (GBC), Light Gradient Boosting Machine (LightGBM), K-Nearest Neighbors (KNN), Decision Tree (DT), Multilayer Perceptron (MLP), Naive Bayes (NB), and Adaptive Boosting (AdaBoost). The area under the receiver operating characteristic (ROC) curve (AUC) served as the primary performance metric. Additional metrics, including sensitivity, specificity, precision, and the F1-score, were used to comprehensively evaluate model accuracy. Calibration curves were generated to assess the consistency between predicted probabilities and observed risk, and the Brier score was used as a quantitative measure of calibration accuracy. Decision curve analysis (DCA) was performed to evaluate the clinical net benefit of each model. To understand the impact of individual predictors on the output, the SHAP method was used to provide transparent insights into each feature’s contribution to the estimated frailty risk. A total of 1,404 participants met the eligibility criteria for this study, of whom 444 (31.62%) were classified as frail. Using the Boruta algorithm and LASSO regression, we identified 10 key predictors of frailty. Among all the ML models tested, the LR model showed the best overall performance, achieving an AUC of 0.759 (95% CI: 0.711–0.806). Shapley Additive exPlanations (SHAP) analysis further revealed the top five predictors of frailty in this population: depression, grip strength, education level, the total number of chronic diseases, and self-rated health. This study introduces an interpretable ML model that effectively detects frailty risk among middle-aged and older adults with GID. The model demonstrates strong predictive accuracy and transparency, supporting its potential as a clinical decision-support tool pending further external validation and real-world deployment. Such proactive measures could improve patient care and promote better long-term health outcomes in this population.

**Supplementary Information:**

The online version contains supplementary material available at 10.1038/s41598-026-50348-x.

## Introduction

Global demographic aging has become a defining trend of our time, making health management for older adults a key focus of geriatric medicine^1^. In this group, gastrointestinal disease (GID) is very common and accounts for a significant part of the worldwide disease burden^2,3^. This includes conditions such as gastritis, gastroesophageal reflux disease (GERD), peptic ulcer disease (PUD), irritable bowel syndrome (IBS), and functional dyspepsia (FD). Beyond reducing patients’ quality of life, GID places a heavy financial burden on healthcare systems worldwide^4^. The prevalence of GID is increasing globally, and the disease causes nearly 8 million deaths each year. Hospitalization rates for these conditions have increased from 4,713 to 5,241 per 100,000 discharges^5^. In the United States, about 24% of the population suffers from GID, with related healthcare costs exceeding $135.9 billion annually^6^. In China, more than 40.7 million outpatient visits involve gastrointestinal symptoms, and 54.4 million visits have a primary diagnosis of GID^7^.

GID is linked not only to local digestive symptoms but also to systemic physiological changes that can lead to frailty. The connection between frailty and GID has been documented^8^. A prospective cohort study in Japan further supported this link, finding a strong correlation between frailty in older adults and gastrointestinal-related quality of life^9^. Furthermore, increasing evidence suggests that specific gastrointestinal disorders, such as IBS ^10^, FD^11^, PUD^12^, and GERD^13^, are significantly associated with frailty or its core components, including sarcopenia and malnutrition.

Frailty is a common condition among older adults, characterized as a state of increased vulnerability to poor resolution of homeostasis following a stressor event^14^. It leads to a gradual decline in multiple physiological systems and functions^15–18^. As a major public health challenge in aging populations, frailty signals a heightened risk for negative health outcomes. Although associated with aging, it can occur independently of chronological age^14^. Frailty affects approximately 7-20% of older adults, with similar rates observed in middle-aged populations^19–21^. Its prevalence increases with age to about 25-50% among those over 85 years old^14^. Prevalence varies by clinical setting: about 10% of community-dwelling older adults are affected^22^, rising to 18-40% in hospitalized patients^23^.

Frailty significantly increases the risk of developing chronic conditions such as diabetes, hypertension, stroke, obesity, and dyslipidemia. This increased risk also leads to worse clinical outcomes, including falls, hospitalization, functional decline, and earlier death, all of which reduce quality of life^24^. Because frailty is dynamic and potentially reversible^25^, early screening of at-risk individuals is crucial to implement effective strategies to prevent or manage the syndrome. However, the high cost of large-scale screening remains a major barrier to its widespread use in routine clinical practice.

Recent advances in machine learning (ML) offer promising alternatives. ML techniques can identify key features in large datasets and help develop predictive models that enhance risk stratification accuracy and facilitate earlier intervention^26^. However, a major challenge for clinical implementation is the “black box” nature of traditional ML models^27^. Interpretable ML, a growing subfield, addresses this issue with frameworks like Shapley Additive exPlanations (SHAP), which is rooted in cooperative game theory. This approach improves model interpretability and accountability^28^ by quantifying each input feature’s contribution and helping identify drivers of frailty risk in middle-aged and older adults with GID. SHAP also offers intuitive visualization tools (such as summary, dependency, and force plots) that translate complex model outputs into clinically useful insights, supporting the real-world application of ML findings.

Although many studies have explored frailty prediction, some using ML techniques in populations like patients with chronic obstructive pulmonary disease (COPD)^29^, research on interpretable ML for predicting frailty risk in individuals with GID remains limited. To address this gap, this study utilized data from the 2013-2015 China Health and Retirement Longitudinal Study (CHARLS) surveys to develop a reliable prediction model for frailty among middle-aged and older adults with GID using explainable ML methods, with the aim of identifying key predictors of frailty, comparing multiple algorithms, and ultimately improving health outcomes and quality of life for this vulnerable group.

## Methods

### Data sources and study design

The data for this analysis were obtained from the China Health and Retirement Longitudinal Study (CHARLS), a nationwide longitudinal survey representing Chinese adults aged 45 and older. CHARLS collects detailed information on demographic characteristics, health status, healthcare utilization, and related expenses. To date, five waves of data have been publicly released (2011, 2013, 2015, 2018, 2020), and their large sample size ensures national representativeness^30^. This study used the 2013 wave as baseline and the 2015 wave for outcome assessment, with a fixed prediction horizon of approximately two years. The study protocol was approved by the Institutional Review Board of Peking University (IRB00001052-11015) in accordance with the Declaration of Helsinki, prior to the enrollment of any participants.

**Fig. 1:**
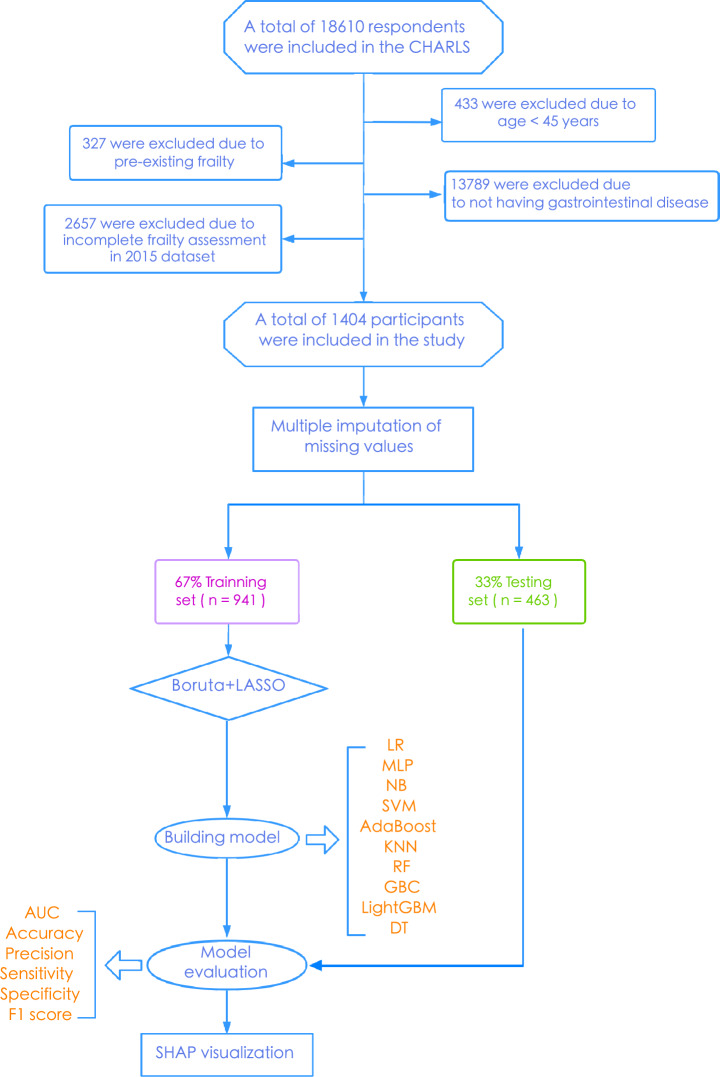
Flow chart of participant selection. LASSO, Least Absolute Shrinkage and Selection Operator; LR, Logistic Regression; MLP, Multilayer Perceptron; NB, Naive Bayes; SVM, Support Vector Machine; AdaBoost, Adaptive Boosting; KNN, k-Nearest Neighbors; RF, Random Forest; GBC, Gradient Boosting Classifier; LightGBM, Light Gradient Boosting Machine; DT, Decision Tree.

Eligibility criteria included: (1) age 45 years or older, and (2) physician-diagnosed GID confirmed by self-report. Exclusion criteria were: (1) baseline frailty in 2013 to ensure the model predicts incident frailty rather than prevalent frailty; (2) inability to provide information independently; (3) non-participation in the 2015 follow-up survey; and (4) incomplete frailty assessment questionnaire in 2015. The final analytical sample consisted of 1,404 middle-aged and older participants. A detailed participant selection flowchart is provided in Fig. 1.

### Assessment instruments and sources

All questionnaires and assessment tools used in this study, including those for frailty, depression, cognitive function, and activities of daily living, were part of the standard China Health and Retirement Longitudinal Study (CHARLS) survey protocol. These instruments have been validated and published in previous studies, as cited in the relevant methodological sections. No new questionnaires were developed specifically for this investigation.

### Assessment of frailty

Frailty was assessed using an adapted version of the Fried frailty phenotype^20^, which has been previously validated in the CHARLS cohort^31,32^. This method defines frailty as a clinical syndrome with five key components: weakness, slowness, exhaustion, reduced physical activity, and weight loss. Frailty status was classified as a dichotomous variable based on the presence of three or more of the following five criteria: (1) Weakness was assessed through self-reported difficulty handling weights over 5 kg^32^; (2) Slowness was identified by reported difficulty in walking 100 meters or climbing multiple flights of stairs without rest^31^; (3) Exhaustion was determined using two CES-D items, where responses of “Most/all of the time” or “Occasionally/moderate amount of time” to either “I felt everything I did was an effort” or “I could not get going” indicated this domain^20^; (4) Reduced physical activity was defined as not engaging in continuous activity or walking for at least 10 minutes during a typical week. Although different from Fried’s original measure, this indicator has been widely used in prior frailty research^31^; (5) Weight loss was identified either by unintentional loss of ≥5 kg over the past year or a current BMI ≤18.5 kg/m^2^.^32^. Unintentional weight loss is regarded as a more accurate marker of frailty than BMI or dietary energy intake measurements^33^.

### Assessment of gastrointestinal disease

GID was identified based on responses from the 2013 baseline health status questionnaire. Participants were asked, “Have you ever been diagnosed by a doctor with stomach or other digestive diseases (excluding tumors or cancer)?” A participant was classified as having GID if they answered “Yes” to this question.

### Assessment of covariates

Demographic and lifestyle data were collected through face-to-face interviews, including age, gender (male/female), marital status (married/cohabiting vs. unmarried), ethnicity (Han vs. non-Han), and residential area (rural vs. urban). Education level was categorized as less than elementary school, elementary school graduate, middle school graduate, or high school graduate and above. Family characteristics included family size, number of surviving children, financial support from parents to children, financial support from children to parents, health insurance coverage, and retirement status. Healthcare utilization measures included outpatient visits (number of visits) in the past month, hospitalizations (number of admissions) in the past year, and length of hospital stay (LOS) in the past year.

Smoking and drinking behaviors were categorized into three groups: never, ever, or current. Participation in social activities was recorded as a yes or no response. Consistent with previous research^34–36^, adequate nightly sleep duration for Chinese adults aged 40 and older was defined as 6–8 hours. Sleep duration was divided into three groups: short (<6 hours), normal (6–8 hours), or long (≥8 hours). Perceived sleep quality was self-reported as good, fair, or poor.

Participants also self-reported chronic medical conditions diagnosed by physicians. These included dyslipidemia, malignancies, cardiovascular diseases, hypertension, stroke, chronic pulmonary diseases, psychiatric disorders, arthritic conditions, renal diseases, asthma, and memory-related disorders such as Alzheimer’s disease, Parkinson’s disease, and cerebral atrophy. The total number of chronic diseases was grouped into four categories: 1, 2, 3, or ≥4. Functional status was assessed using the Activities of Daily Living (ADL) and Instrumental Activities of Daily Living (IADL) scales^37^. Chronic pain was defined as pain lasting longer than the expected healing period or persisting despite the absence of underlying tissue damage^38^. Other physical indicators included edentulism (complete tooth loss), visual impairment (VI), and hearing impairment (HI): VI was defined by self-reported “fair” or “poor” distance or near vision (with corrective lenses if used), and HI by self-reported “fair” or “poor” hearing (with hearing aids if used)^39^.

Mental health factors included hope for the future (yes/no), life satisfaction (good/fair/poor), self-rated health (good/fair/poor), and depression. Depressive symptoms were assessed using the 10-item CES-D (CES-D-10), with responses scored on a 4-point scale (0 = rarely, <2 days; 3 = most days, 5–7 days) and a total score ranging from 0 to 30 (higher scores indicate more severe symptoms). A cutoff score of ≥10 was used to define depression, which has been validated in Chinese adults aged ≥45 years^41,42^.

Cognitive function was assessed using two validated tools. The mental status assessment (range 0–11) measured orientation, visuospatial skills, and numerical ability through a serial-7 subtraction task and recorded whether additional explanation was needed during testing. This tool is comparable to the Telephone Interview of Cognitive Status (TICS-10)^43^. Additionally, a memory score (range 0–10) was calculated from the average of immediate and delayed recall of 10 Chinese words, with higher scores indicating better episodic memory function^44^.

### Missing value processing

Missing data are common in CHARLS, and excluding participants with incomplete data may cause selection bias, which can reduce sample representativeness and generalizability. To address this, variables with >30% missing data were first excluded, and missing data in the remaining variables were imputed using multiple imputation by chained equations (MICE) in R. Imputation was performed using predictive mean matching for continuous variables and logistic regression for binary variables, generating five imputed datasets. To prevent information leakage, imputation was conducted before data splitting. Model performance metrics were pooled across the five imputed datasets using Rubin’s rules.

### Feature selection

Feature selection was performed through a two-step process combining the Boruta algorithm with LASSO regression. The Boruta method creates randomly shuffled “shadow features” for each original predictor and builds a random forest model with this expanded set. It assesses feature relevance by comparing the importance of the real features to their shuffled counterparts, labeling those with significantly higher importance scores as confirmed predictors. Features close to the threshold were temporarily retained^45,46^. Next, LASSO regression further refined the feature set. LASSO applies an L1 penalty to the least-squares loss function, shrinking the coefficients of less relevant predictors toward zero. The regularization parameter λ (λ ≥ 0) was determined using 10-fold cross-validation. In this process, the dataset was randomly divided into 10 non-overlapping groups. Each iteration involved training the model on nine groups to test different λ values, with the remaining group used for validation. This was repeated 10 times, allowing each group to serve as the test set once. The optimal λ was selected based on the lowest mean squared error (MSE). Typically, two options are considered for the best λ: λ.min, which results in the lowest cross-validation error, and λ.1se, which produces a simpler model with a prediction error within 1 standard error of the minimum. The choice is guided by the study’s context and objectives. Additionally, LASSO helps reduce multicollinearity by shrinking the coefficients of irrelevant predictors^47^.

### Model construction and evaluation

ML techniques are generally categorized into four main types: supervised, unsupervised, semi-supervised, and reinforcement learning. Since this study aims to classify subjects into two distinct categories (frail vs. non-frail), this constitutes a binary classification problem. As a result, supervised learning methods were deemed most appropriate and were used in this research^48^. Ten standard supervised learning algorithms were employed: Logistic Regression (LR), Multilayer Perceptron (MLP), Naive Bayes (NB), Support Vector Machine (SVM), Adaptive Boosting (AdaBoost), K-Nearest Neighbors (KNN), Random Forest (RF), Gradient Boosting Classifier (GBC), Light Gradient Boosting Machine (LightGBM), and Decision Tree (DT).

Data were randomly split into a training set (67%) for model building and a held-out testing set (33%) for final evaluation. 10-fold cross-validation on the training set was used for hyperparameter tuning and model selection. After tuning, each algorithm was retrained on the entire training set and then evaluated on the held-out testing set. The Synthetic Minority Oversampling Technique (SMOTE) was applied only to the training set to address class imbalance, ensuring the testing set remained independent and unbiased for estimating the model’s generalizability. Hyperparameter tuning was performed using grid search with 10-fold cross-validation on the training set. The optimal hyperparameters for each algorithm were selected based on the highest cross-validated AUC. The optimal parameters for all algorithms are summarized in Supplementary Table 1.

Model performance was evaluated along three core dimensions: discriminative ability, calibration ability, and clinical utility. Internal validation was performed using the held-out testing set. Discrimination, which reflects how well the model separates outcome classes, was quantified using metrics including the area under the receiver operating characteristic (ROC) curve (AUC), accuracy, sensitivity, specificity, precision, and the F1-score. Calibration was evaluated by comparing predicted probabilities with observed outcomes using calibration curves, with the Brier score as a quantitative measure of overall calibration accuracy. Clinical value was measured using decision curve analysis (DCA), which estimates the net benefit of each model at different risk thresholds.

### Evaluation of the importance of variables

To evaluate predictor importance, we used the SHAP framework, a game-theoretic approach for interpreting ML outputs^49^. SHAP values quantify the marginal contribution of each feature to the model’s prediction for each observation, enabling the interpretation of complex model behavior^49^. In this analysis, positive SHAP values indicate an increased probability of frailty, while negative values indicate a decreased risk of the syndrome. SHAP calculations were performed using the KernelExplainer for model-agnostic interpretation. To visualize the results, we used beeswarm plots to illustrate feature importance and the direction of their effects, bar plots to display mean absolute SHAP values as a global measure of feature importance, and force plots and waterfall plots to explain individual-level predictions. This approach to SHAP-based model explanation has been increasingly used in clinical research to improve transparency and identify key predictors from population health data^50,51^. This framework enhances the interpretability of the ML model, addressing the “black box” limitation of traditional algorithms.

### Correlation analysis

Correlation analysis was conducted after feature selection to examine the interrelationships among the final predictors. This analysis was purely descriptive and did not influence the feature selection strategy or the final set of predictors. Pearson correlation coefficients were calculated for continuous variables, while Spearman’s rank correlation was used for ordinal variables.

### Statistical analysis

For continuous variables, data are presented as mean ± standard deviation (SD); for categorical variables, data are shown as counts (n) and percentages (%). Between-group comparisons used independent-samples t-tests for normally distributed continuous variables, the Mann-Whitney U test for nonparametric continuous data, and the chi-square test for categorical variables. All dichotomous variables were coded as 1 (“yes”) / 0 (“no”). Statistical analyses were performed using R (http://www.R-project.org) and Free Statistics software version 2.1. SHAP analysis was conducted using the Python shap library (version 0.41.0) with the KernelExplainer. A two-sided p < 0.05 was considered statistically significant.

## Results

### Subject characteristics

The ML prediction models were developed using data from 1,404 middle-aged and older adults diagnosed with GID. Among them, 444 individuals were classified as frail, accounting for 31.62% of the cohort (Table 1). Frail individuals differed significantly from non-frail individuals. They tended to be older, female, unmarried, and living in rural areas; had lower educational attainment (below elementary level); and had more surviving children. Additionally, they were more likely to have had outpatient visits in the past month, a higher number of outpatient visits, hospitalization in the past year, more frequent hospital admissions, and longer LOS. They were also more likely to have never smoked or consumed alcohol, not participate in social activities, and not be retired. They tended to have shorter sleep duration, poorer sleep quality, chronic pain, poorer self-rated health, lower grip strength, lower life satisfaction, greater uncertainty about the future, depression, lower cognitive function, and higher ADL and IADL scores. They also had a history of falls, VI, HI, edentulism, and four or more chronic comorbidities.

**Table 1:** Baseline characteristics of the participants.

**Variables**	**Total (n = 1404)**	**Non-frail** **(n = 960)**	**Frail** **(n = 444)**		***p*-Value**
**Age (years), Mean ± SD**	59.4 ± 8.9	58.6 ± 8.7		61.2 ± 9.1	< 0.001
**Gender, n (%)**					< 0.001
Female	829 (59.0)	509 (53)		320 (72.1)	
Male	575 (41.0)	451 (47)		124 (27.9)	
**Ethnicity, n (%)**					0.332
Non-Han	142 (10.1)	92 (9.6)		50 (11.3)	
Han	1262 (89.9)	868 (90.4)		394 (88.7)	
**Marital status, n (%)**					0.861
Unmarried	158 (11.3)	109 (11.4)		49 (11)	
Married/Cohabitated	1246 (88.7)	851 (88.6)		395 (89)	
**Area of residence, n (%)**					< 0.001
Urban	453 (32.3)	341 (35.5)		112 (25.2)	
Rural	951 (67.7)	619 (64.5)		332 (74.8)	
**Education level, n (%)**					< 0.001
Below elementary school	711 (50.6)	427 (44.5)		284 (64)	
Elementary school graduate	316 (22.5)	227 (23.6)		89 (20)	
Middle school graduate	263 (18.7)	211 (22)		52 (11.7)	
High school graduate and above	114 (8.1)	95 (9.9)		19 (4.3)	
**Insurance, n (%)**					0.735
None	32 (2.3)	21 (2.2)		11 (2.5)	
Yes	1372 (97.7)	939 (97.8)		433 (97.5)	
**Cfsfp, Median (IQR)**	1223.0 (50.0, 4000.0)	1210.0 (0.0, 4000.0)		1255.0 (100.0, 3812.5)	0.914
**Pfsfc, Median (IQR)**	0.0 (0.0, 700.0)	0.0 (0.0, 925.0)		0.0 (0.0, 500.0)	0.172
**Family size, Mean ± SD**	3.7 ± 1.8	3.7 ± 1.8		3.6 ± 1.8	0.764
**The number of Surviving Children, Mean ± SD**	2.8 ± 1.5	2.7 ± 1.4		3.0 ± 1.5	< 0.001
**Outpatient Visit, n (%)**					0.002
No	1001 (71.3)	709 (73.9)		292 (65.8)	
Yes	403 (28.7)	251 (26.1)		152 (34.2)	
**The number of Outpatient Visits, Median (IQR)**	0.0 (0.0, 1.0)	0.0 (0.0, 1.0)		0.0 (0.0, 1.0)	< 0.001
**Hospitalization, n (%)**					< 0.001
No	1167 (83.1)	835 (87)		332 (74.8)	
Yes	237 (16.9)	125 (13)		112 (25.2)	
**The number of Hospitalizations, Median (IQR)**	0.0 (0.0, 0.0)	0.0 (0.0, 0.0)		0.0 (0.0, 1.0)	< 0.001
**LOS, Median (IQR)**	0.0 (0.0, 0.0)	0.0 (0.0, 0.0)		0.0 (0.0, 0.0)	< 0.001
**Smoking, n (%)**					< 0.001
Never	866 (61.7)	546 (56.9)		320 (72.1)	
Ever	181 (12.9)	141 (14.7)		40 (9)	
Current	357 (25.4)	273 (28.4)		84 (18.9)	
**Drinking, n (%)**					0.005
Never	816 (58.1)	530 (55.2)		286 (64.4)	
Ever	176 (12.5)	127 (13.2)		49 (11)	
Current	412 (29.3)	303 (31.6)		109 (24.5)	
**Retire, n (%)**					0.01
No	1278 (91.0)	861 (89.7)		417 (93.9)	
Yes	126 (9.0)	99 (10.3)		27 (6.1)	
**Social activity, n (%)**					< 0.001
None	649 (46.2)	406 (42.3)		243 (54.7)	
Yes	755 (53.8)	554 (57.7)		201 (45.3)	
**Sleep duration, n (%)**					< 0.001
Short sleep	617 (43.9)	379 (39.5)		238 (53.6)	
Normal sleep	520 (37.0)	387 (40.3)		133 (30)	
Long sleep	267 (19.0)	194 (20.2)		73 (16.4)	
**Sleep quality, n (%)**					< 0.001
Good	549 (39.1)	432 (45)		117 (26.4)	
Fair	458 (32.6)	310 (32.3)		148 (33.3)	
Poor	397 (28.3)	218 (22.7)		179 (40.3)	
**Chronic pain, n (%)**					< 0.001
No	683 (48.6)	533 (55.5)		150 (33.8)	
Yes	721 (51.4)	427 (44.5)		294 (66.2)	
**Grip strength (kg), Mean ± SD**	31.7 ± 9.8	33.2 ± 9.9		28.3 ± 8.9	< 0.001
**Waistline (cm), Mean ± SD**	85.1 ± 9.8	85.2 ± 9.1		84.8 ± 11.2	0.484
**Self-rated health, n (%)**					< 0.001
Good	157 (11.2)	127 (13.2)		30 (6.8)	
Fair	739 (52.6)	560 (58.3)		179 (40.3)	
Poor	508 (36.2)	273 (28.4)		235 (52.9)	
**Life satisfaction, n (%)**					< 0.001
Good	299 (21.3)	208 (21.7)		91 (20.5)	
Fair	865 (61.6)	624 (65)		241 (54.3)	
Poor	240 (17.1)	128 (13.3)		112 (25.2)	
**Hope, n (%)**					0.042
No	1009 (71.9)	674 (70.2)		335 (75.5)	
Yes	395 (28.1)	286 (29.8)		109 (24.5)	
**Depression, n (%)**					< 0.001
No	777 (55.3)	620 (64.6)		157 (35.4)	
Yes	627 (44.7)	340 (35.4)		287 (64.6)	
**Cognitive function, Mean ± SD**	11.6 ± 3.2	11.9 ± 3.1		10.8 ± 3.1	< 0.001
**ADL score, Median (IQR)**	0.0 (0.0, 0.0)	0.0 (0.0, 0.0)		0.0 (0.0, 1.0)	< 0.001
**IADL score, Median (IQR)**	0.0 (0.0, 0.0)	0.0 (0.0, 0.0)		0.0 (0.0, 1.0)	< 0.001
**Fall down, n (%)**					< 0.001
No	1110 (79.1)	791 (82.4)		319 (71.8)	
Yes	294 (20.9)	169 (17.6)		125 (28.2)	
**Hip fracture, n (%)**					1
No	1395 (99.4)	954 (99.4)		441 (99.3)	
Yes	9 (0.6)	6 (0.6)		3 (0.7)	
**VI, n (%)**					< 0.001
No	1186 (84.5)	786 (81.9)		400 (90.1)	
Yes	218 (15.5)	174 (18.1)		44 (9.9)	
**HI, n (%)**					0.006
No	1195 (85.1)	800 (83.3)		395 (89)	
Yes	209 (14.9)	160 (16.7)		49 (11)	
**Edentulism, n (%)**					< 0.001
No	1227 (87.4)	862 (89.8)		365 (82.2)	
Yes	177 (12.6)	98 (10.2)		79 (17.8)	
**The total number of chronic diseases, n (%)**					< 0.001
1	332 (23.6)	265 (27.6)		67 (15.1)	
2	430 (30.6)	309 (32.2)		121 (27.3)	
3	287 (20.4)	194 (20.2)		93 (20.9)	
≥4	355 (25.3)	192 (20)		163 (36.7)	

Cfsfp: Children’s financial support for parents; Pfsfc: Parents’ financial support for their children; LOS, Length of Hospital Stay; IADL, Instrumental Activities of Daily Living; ADL, Activity of Daily Living; VI, Visual impairment; HI, Hearing impairment.

Additionally, to evaluate the suitability of the data partitioning, we compared baseline characteristics between the training and testing sets; the full results are shown in Table 2. All variables were well balanced (all P > 0.05), confirming the success of the partition and supporting the reliability of subsequent analytical procedures.

**Table 2:** Comparison of variables between the training set and the testing set of middle-aged and older patients with gastrointestinal disease.

**Variables**	**Total (n = 1404)**	**Training set (n = 941)**	**Testing set (n = 463)**		***p*-Value**
**Age (years), Mean ± SD**	59.4 ± 8.9	59.5 ± 8.8		59.2 ± 9.1	0.522
**Gender, n (%)**					0.394
Female	829 (59.0)	563 (59.8)		266 (57.5)	
Male	575 (41.0)	378 (40.2)		197 (42.5)	
**Ethnicity, n (%)**					0.363
Non-Han	142 (10.1)	100 (10.6)		42 (9.1)	
Han	1262 (89.9)	841 (89.4)		421 (90.9)	
**Marital status, n (%)**					0.461
Unmarried	158 (11.3)	110 (11.7)		48 (10.4)	
Married/Cohabitated	1246 (88.7)	831 (88.3)		415 (89.6)	
**Area of residence, n (%)**					0.575
Urban	453 (32.3)	299 (31.8)		154 (33.3)	
Rural	951 (67.7)	642 (68.2)		309 (66.7)	
**Education level, n (%)**					0.924
Below elementary school	711 (50.6)	479 (50.9)		232 (50.1)	
Elementary school graduate	316 (22.5)	207 (22)		109 (23.5)	
Middle school graduate	263 (18.7)	177 (18.8)		86 (18.6)	
High school graduate and above	114 (8.1)	78 (8.3)		36 (7.8)	
**Insurance, n (%)**					0.833
None	32 (2.3)	22 (2.3)		10 (2.2)	
Yes	1372 (97.7)	919 (97.7)		453 (97.8)	
**Cfsfp, Median (IQR)**	1223.0 (50.0, 4000.0)	1200.0 (100.0, 3900.0)		1232.0 (0.0, 4155.0)	0.863
**Pfsfc, Median (IQR)**	0.0 (0.0, 700.0)	0.0 (0.0, 750.0)		0.0 (0.0, 680.0)	0.326
**Family size, Mean ± SD**	3.7 ± 1.8	3.6 ± 1.8		3.8 ± 1.9	0.168
**The number of Surviving Children, Mean ± SD**	2.8 ± 1.5	2.8 ± 1.4		2.8 ± 1.5	0.802
**Outpatient Visit, n (%)**					0.214
No	1001 (71.3)	661 (70.2)		340 (73.4)	
Yes	403 (28.7)	280 (29.8)		123 (26.6)	
**The number of Outpatient Visits, Median (IQR)**	0.0 (0.0, 1.0)	0.0 (0.0, 1.0)		0.0 (0.0, 1.0)	0.128
**Hospitalization, n (%)**					0.124
No	1167 (83.1)	772 (82)		395 (85.3)	
Yes	237 (16.9)	169 (18)		68 (14.7)	
**The number of Hospitalizations, Median (IQR)**	0.0 (0.0, 0.0)	0.0 (0.0, 0.0)		0.0 (0.0, 0.0)	0.096
**LOS, Median (IQR)**	0.0 (0.0, 0.0)	0.0 (0.0, 0.0)		0.0 (0.0, 0.0)	0.109
**Smoking, n (%)**					0.543
Never	866 (61.7)	587 (62.4)		279 (60.3)	
Ever	181 (12.9)	115 (12.2)		66 (14.3)	
Current	357 (25.4)	239 (25.4)		118 (25.5)	
**Drinking, n (%)**					0.151
Never	816 (58.1)	543 (57.7)		273 (59)	
Ever	176 (12.5)	129 (13.7)		47 (10.2)	
Current	412 (29.3)	269 (28.6)		143 (30.9)	
**Retire, n (%)**					0.758
No	1278 (91.0)	855 (90.9)		423 (91.4)	
Yes	126 (9.0)	86 (9.1)		40 (8.6)	
**Social activity, n (%)**					0.361
None	649 (46.2)	443 (47.1)		206 (44.5)	
Yes	755 (53.8)	498 (52.9)		257 (55.5)	
**Sleep duration, n (%)**					0.665
Short sleep	617 (43.9)	407 (43.3)		210 (45.4)	
Normal sleep	520 (37.0)	356 (37.8)		164 (35.4)	
Long sleep	267 (19.0)	178 (18.9)		89 (19.2)	
**Sleep quality, n (%)**					0.933
Good	549 (39.1)	366 (38.9)		183 (39.5)	
Fair	458 (32.6)	306 (32.5)		152 (32.8)	
Poor	397 (28.3)	269 (28.6)		128 (27.6)	
**Chronic pain, n (%)**					0.8
No	683 (48.6)	460 (48.9)		223 (48.2)	
Yes	721 (51.4)	481 (51.1)		240 (51.8)	
**Grip strength (kg), Mean ± SD**	31.7 ± 9.8	31.5 ± 9.6		32.0 ± 10.4	0.395
**Waistline (cm), Mean ± SD**	85.1 ± 9.8	85.2 ± 9.6		84.8 ± 10.1	0.398
**Self-rated health, n (%)**					0.257
Good	157 (11.2)	110 (11.7)		47 (10.2)	
Fair	739 (52.6)	481 (51.1)		258 (55.7)	
Poor	508 (36.2)	350 (37.2)		158 (34.1)	
**Life satisfaction, n (%)**					0.204
Good	299 (21.3)	189 (20.1)		110 (23.8)	
Fair	865 (61.6)	594 (63.1)		271 (58.5)	
Poor	240 (17.1)	158 (16.8)		82 (17.7)	
**Hope, n (%)**					0.974
No	1009 (71.9)	676 (71.8)		333 (71.9)	
Yes	395 (28.1)	265 (28.2)		130 (28.1)	
**Depression, n (%)**					0.44
No	777 (55.3)	514 (54.6)		263 (56.8)	
Yes	627 (44.7)	427 (45.4)		200 (43.2)	
**Cognitive function, Mean ± SD**	11.6 ± 3.2	11.5 ± 3.1		11.7 ± 3.3	0.394
**ADL score, Median (IQR)**	0.0 (0.0, 0.0)	0.0 (0.0, 0.0)		0.0 (0.0, 0.0)	0.107
**IADL score, Median (IQR)**	0.0 (0.0, 0.0)	0.0 (0.0, 1.0)		0.0 (0.0, 0.0)	0.152
**Fall down, n (%)**					0.052
No	1110 (79.1)	730 (77.6)		380 (82.1)	
Yes	294 (20.9)	211 (22.4)		83 (17.9)	
**Hip fracture, n (%)**					0.726
No	1395 (99.4)	934 (99.3)		461 (99.6)	
Yes	9 (0.6)	7 (0.7)		2 (0.4)	
**VI, n (%)**					0.626
No	1186 (84.5)	798 (84.8)		388 (83.8)	
Yes	218 (15.5)	143 (15.2)		75 (16.2)	
**HI, n (%)**					0.864
No	1195 (85.1)	802 (85.2)		393 (84.9)	
Yes	209 (14.9)	139 (14.8)		70 (15.1)	
**Edentulism, n (%)**					0.685
No	1227 (87.4)	820 (87.1)		407 (87.9)	
Yes	177 (12.6)	121 (12.9)		56 (12.1)	
**The total number of chronic diseases, n (%)**					0.194
1	332 (23.6)	214 (22.7)		118 (25.5)	
2	430 (30.6)	282 (30)		148 (32)	
3	287 (20.4)	191 (20.3)		96 (20.7)	
≥4	355 (25.3)	254 (27)		101 (21.8)	
**Frailty, n (%)**					0.576
No	960 (68.4)	648 (68.9)		312 (67.4)	
Yes	444 (31.6)	293 (31.1)		151 (32.6)	

Cfsfp: Children’s financial support for parents; Pfsfc: Parents’ financial support for their children; LOS, Length of Hospital Stay; IADL, Instrumental Activities of Daily Living; ADL, Activity of Daily Living; VI, Visual impairment; HI, Hearing impairment.

### Feature selection

**Fig. 2:**
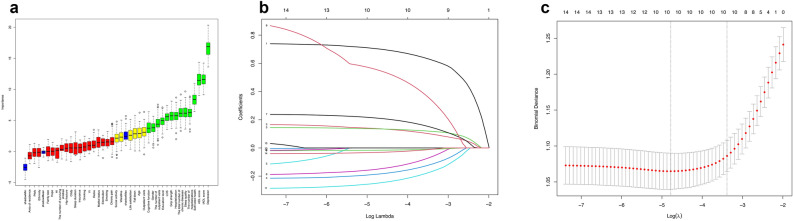
Feature selection using the Boruta algorithm and LASSO regression. (**a**) Variable importance assessment by the Boruta algorithm. Features are classified as confirmed predictors (green), tentative predictors (yellow), or irrelevant variables (red) based on their significance compared to shadow features. (**b**) Coefficient trajectories of the 14 variables retained from the Boruta analysis across different regularization parameters (log(λ)) in LASSO regression. (**c**) 10-fold cross-validation curve for LASSO. The left dashed line (λ.min) indicates the λ value with minimum cross-validation error, and the right dashed line (λ.1se) corresponds to the most regularized model within one standard error of the minimum. LASSO, Least Absolute Shrinkage and Selection Operator.

Initial variable screening using the Boruta algorithm (Fig. 2a) identified essential predictors of frailty in this population: depression, ADL score, IADL score, self-rated health, the number of hospitalizations, sleep quality, the total number of chronic diseases, LOS, hospitalization, grip strength, education level, the number of outpatient visits, cognitive function, and gender. Next, LASSO regression was applied to refine the variables using 10-fold cross-validation for parameter tuning. Fig. 2b illustrates the variable selection process, where each curve shows the coefficient path of a variable as the regularization intensity increases. Variables with greater importance tend to remain non-zero until stronger regularization is applied. Fig. 2c displays two vertical dashed lines indicating the values of λ.min and λ.1se, which correspond to the optimal and most parsimonious models, respectively. Whether λ.min or λ.1se was chosen, LASSO consistently selected 10 key variables: depression, ADL score, IADL score, self-rated health, sleep quality, the total number of chronic diseases, hospitalization, grip strength, education level, and cognitive function, resulting in a simplified predictive model.

### Evaluation of model performance

**Fig. 3:**
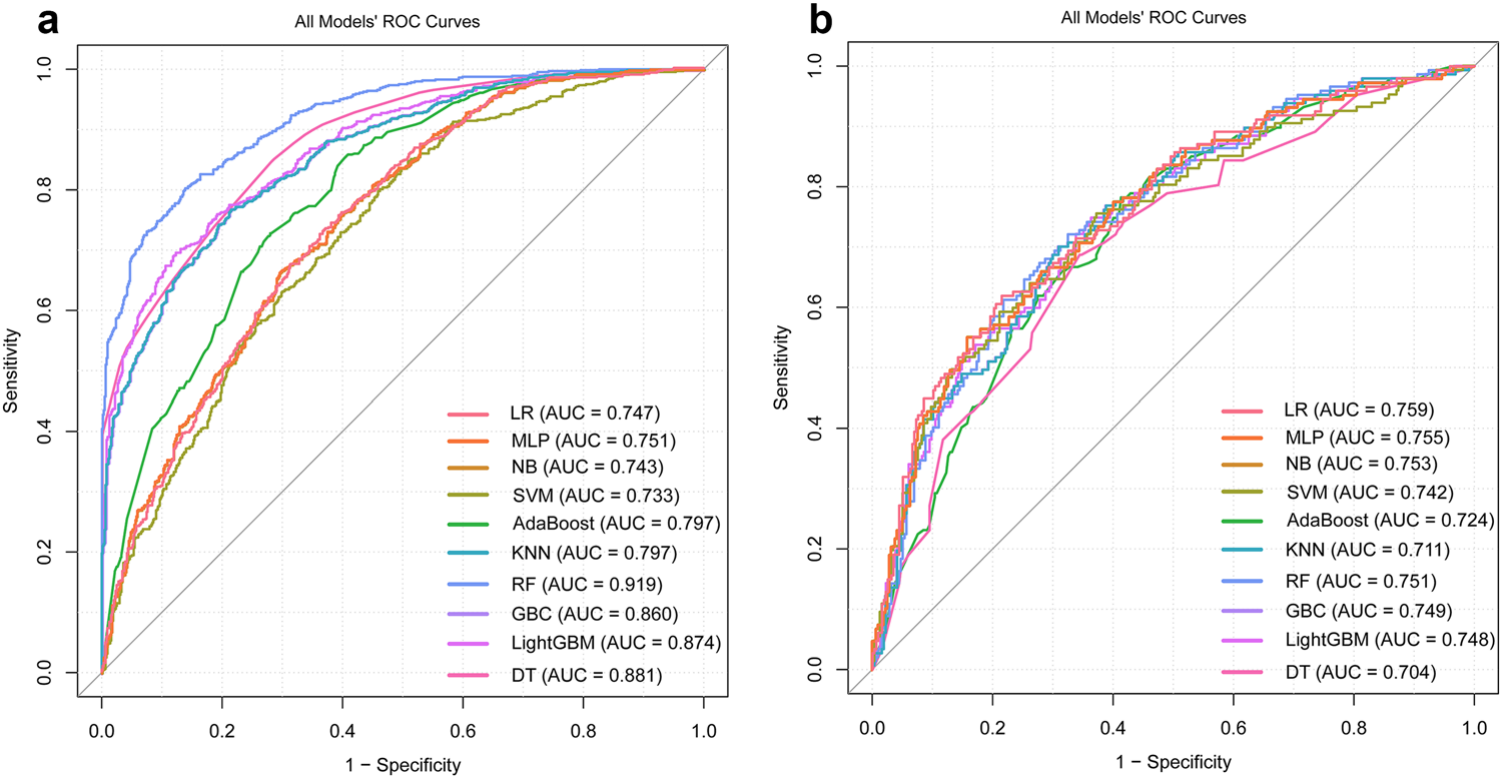
ROC curves for the ten machine learning models in the training set (**a**) and testing set (**b**). LR, Logistic Regression; MLP, Multilayer Perceptron; NB, Naive Bayes; SVM, Support Vector Machine; AdaBoost, Adaptive Boosting; KNN, k-Nearest Neighbors; RF, Random Forest; GBC, Gradient Boosting Classifier; LightGBM, Light Gradient Boosting Machine; DT, Decision Tree.

**Fig. 4:**
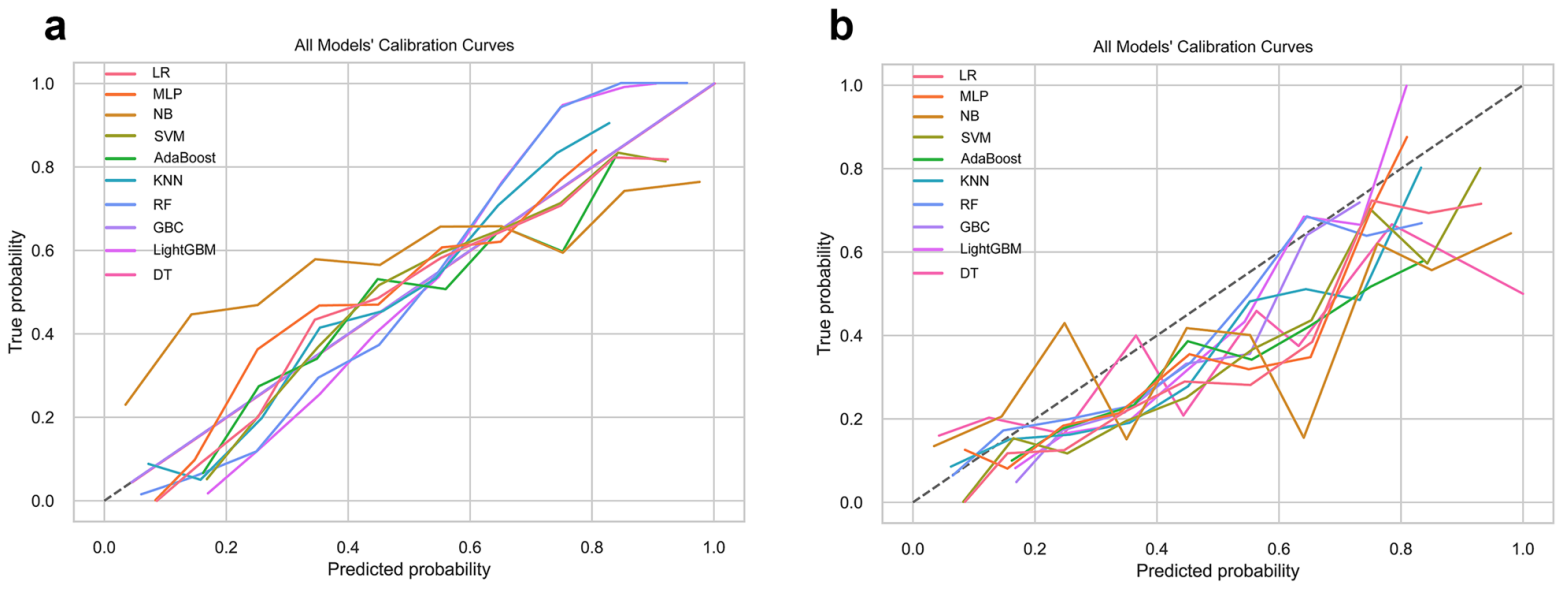
Calibration curves for the ten machine learning models in the training set (**a**) and testing set (**b**). The diagonal dashed line represents perfect calibration, where predicted probabilities equal observed event rates. Curves closer to this line indicate better calibration. LR, Logistic Regression; MLP, Multilayer Perceptron; NB, Naive Bayes; SVM, Support Vector Machine; AdaBoost, Adaptive Boosting; KNN, k-Nearest Neighbors; RF, Random Forest; GBC, Gradient Boosting Classifier; LightGBM, Light Gradient Boosting Machine; DT, Decision Tree.

**Fig. 5:**
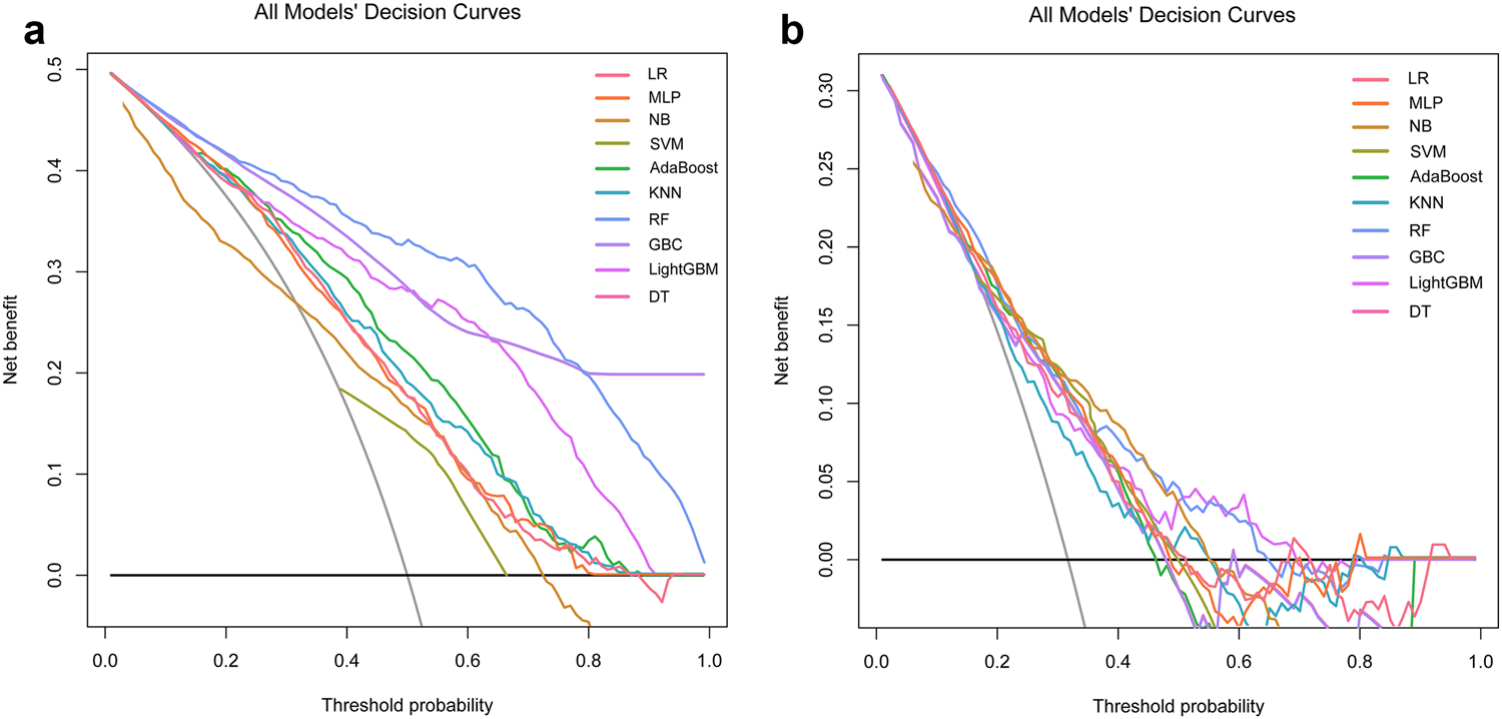
Decision curves for the ten machine learning models in the training set (**a**) and testing set (**b**). The y-axis represents net benefit, and the x-axis represents threshold probability. Curves above the “Treat all” and “Treat none” lines indicate clinical utility. LR, Logistic Regression; MLP, Multilayer Perceptron; NB, Naive Bayes; SVM, Support Vector Machine; AdaBoost, Adaptive Boosting; KNN, k-Nearest Neighbors; RF, Random Forest; GBC, Gradient Boosting Classifier; LightGBM, Light Gradient Boosting Machine; DT, Decision Tree.

Ten ML models were built and evaluated on both the training and testing datasets for internal validation. The corresponding ROC curves are presented in Fig. 3a (training) and Fig. 3b (testing). Performance metrics, including AUC, accuracy, sensitivity, specificity, precision, F1-score, and Brier score, are provided in Tables 3 and 4. During training, the RF model achieved the highest AUC (0.919), followed by the DT model (AUC=0.881), LightGBM model (AUC=0.874), GBC model (AUC=0.860), AdaBoost model (AUC=0.797), KNN model (AUC=0.772), MLP model (AUC=0.751), LR model (AUC=0.747), NB model (AUC=0.743), and SVM model (AUC=0.733). When assessed on the testing set, the LR model showed superior performance with an AUC of 0.759 (95% CI: 0.711–0.806), outperforming the MLP model (AUC=0.755), NB model (AUC=0.753), RF model (AUC=0.751), GBC model (AUC=0.749), LightGBM model (AUC=0.748), SVM model (AUC=0.742), AdaBoost model (AUC=0.724), KNN model (AUC=0.711) and DT model (AUC=0.704). The RF, GBC, LightGBM, and DT models were excluded from the comparative analysis due to the risk of overfitting. Among the remaining six models, the NB model attained the highest scores in accuracy (0.718), specificity (0.782), and precision (0.552). The SVM model showed the highest sensitivity (0.844), and the LR model obtained the best F1 score (0.576). Additionally, the LR model showed the most balanced performance across all evaluation metrics, with an accuracy of 0.685, sensitivity of 0.673, specificity of 0.691, and precision of 0.503. Calibration curves for all models are presented in Fig. 4a (training) and Fig. 4b (testing). The Brier score, which quantifies calibration accuracy, is reported in Tables 3 and 4. The LR model achieved a Brier score of 0.204 in the training set and 0.200 in the testing set, further supporting its good calibration. Of the models assessed, the LR model demonstrated the closest alignment with the reference line in these plots, indicating highly consistent calibration between its risk predictions and observed event rates in both datasets. Further supporting its clinical value, DCA (Figs. 5a and 5b) showed that the LR model offered superior net benefits across most decision thresholds.

**Table 3:** Comparison of the predictive ability of several models in the training set.

**Model**	**AUC**	**Accuracy**	**Sensitivity**	**Specificity**	**Precision**	**F1**	**Brier**
**LR**	0.747	0.676	0.680	0.672	0.674	0.677	0.204
**MLP**	0.751	0.677	0.686	0.667	0.673	0.680	0.203
**NB**	0.743	0.666	0.566	0.765	0.707	0.629	0.247
**SVM**	0.733	0.659	0.869	0.449	0.612	0.719	0.218
**AdaBoost**	0.797	0.719	0.762	0.677	0.702	0.731	0.215
**KNN**	0.772	0.690	0.698	0.681	0.687	0.692	0.196
**RF**	0.919	0.830	0.826	0.835	0.834	0.830	0.125
**GBC**	0.860	0.769	0.782	0.756	0.762	0.772	0.163
**LightGBM**	0.874	0.780	0.767	0.793	0.788	0.777	0.158
**DT**	0.881	0.783	0.846	0.720	0.751	0.796	0.174

LR, Logistic Regression; MLP, Multilayer Perceptron; NB, Naive Bayes; SVM, Support Vector Machine; Ada Boost, Adaptive Boosting; KNN, k-Nearest Neighbors; RF, Random Forest; GBC, Gradient Boosting Classifier; LightGBM, Light Gradient Boosting Machine; DT, Decision Tree.

**Table 4:** Comparison of the predictive power of several models in the testing set.

**Model**	**AUC**	**Accuracy**	**Sensitivity**	**Specificity**	**Precision**	**F1**	**Brier**
**LR**	0.759	0.685	0.673	0.691	0.503	0.576	0.200
**MLP**	0.755	0.672	0.673	0.672	0.488	0.566	0.197
**NB**	0.753	0.718	0.578	0.782	0.552	0.565	0.213
**SVM**	0.742	0.573	0.844	0.448	0.415	0.556	0.192
**AdaBoost**	0.724	0.664	0.667	0.662	0.478	0.557	0.186
**KNN**	0.711	0.696	0.612	0.735	0.517	0.561	0.207
**RF**	0.751	0.728	0.578	0.798	0.570	0.574	0.184
**GBC**	0.749	0.705	0.585	0.760	0.531	0.557	0.191
**LightGBM**	0.748	0.720	0.565	0.792	0.557	0.561	0.191
**DT**	0.704	0.666	0.687	0.656	0.481	0.566	0.217

LR, Logistic Regression; MLP, Multilayer Perceptron; NB, Naive Bayes; SVM, Support Vector Machine; Ada Boost, Adaptive Boosting; KNN, k-Nearest Neighbors; RF, Random Forest; GBC, Gradient Boosting Classifier; LightGBM, Light Gradient Boosting Machine; DT, Decision Tree.

The LR model was selected as the final model based on a combination of discriminative performance (AUC), calibration (the Brier score, calibration curves), and clinical utility (DCA). Although tree-based models (RF, GBC, LightGBM, DT) achieved higher AUC on the training set, they showed evidence of overfitting, with substantially lower performance on the testing set, and were therefore excluded from the final selection.

### Evaluation of the importance of variables

**Fig. 6:**
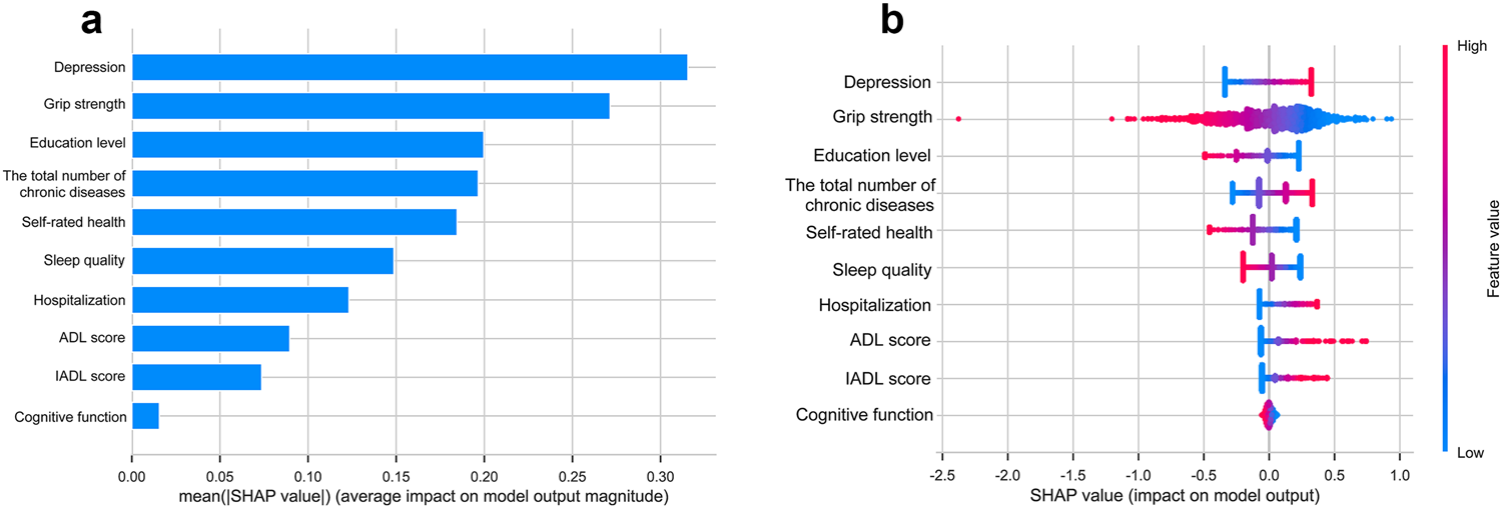
Interpretability of the LR model using SHAP. (**a**) Bar plot of mean absolute SHAP values, ranking predictors by their overall importance in the model. (**b**) Beeswarm plot of SHAP values, showing the impact and direction of effect for each predictor. Each point represents an individual participant. The color indicates the feature value (red: high; blue: low). Points to the right of the vertical line (SHAP value = 0) increase frailty risk; points to the left decrease risk. LR, Logistic Regression; SHAP, Shapley Additive exPlanations.

We used the SHAP approach to analyze the contributions of predictors in the LR model and summarized the findings in Fig. 6. SHAP values quantify both the magnitude and direction of each variable’s impact on predictions, with larger absolute values indicating greater influence^51^. Figure 6a shows variables ordered from top to bottom in ascending order of their contribution to frailty prediction, with the vertical axis marked by an SHAP value of 0. Red points to the right of this axis indicate a positive contribution to the predicted outcome (i.e., increased frailty risk). In contrast, blue points to the right of the axis indicate a negative contribution (i.e., decreased frailty risk). Figure 6b displays the ranking of predictor variables by their mean absolute SHAP values, with feature importance decreasing from top to bottom. The five strongest determinants of frailty in this population, ranked from most to least important, were depression, grip strength, education level, the total number of chronic diseases, and self-rated health. Specifically, both depression and a greater number of chronic conditions showed positive associations with frailty, suggesting that GID patients with either condition face increased frailty risk. Conversely, grip strength, education level, and self-rated health showed inverse relationships with frailty. Specifically, weaker grip strength, lower education, and poorer self-rated health were all associated with a higher probability of frailty.

### Clinical application of models

**Fig. 7:**
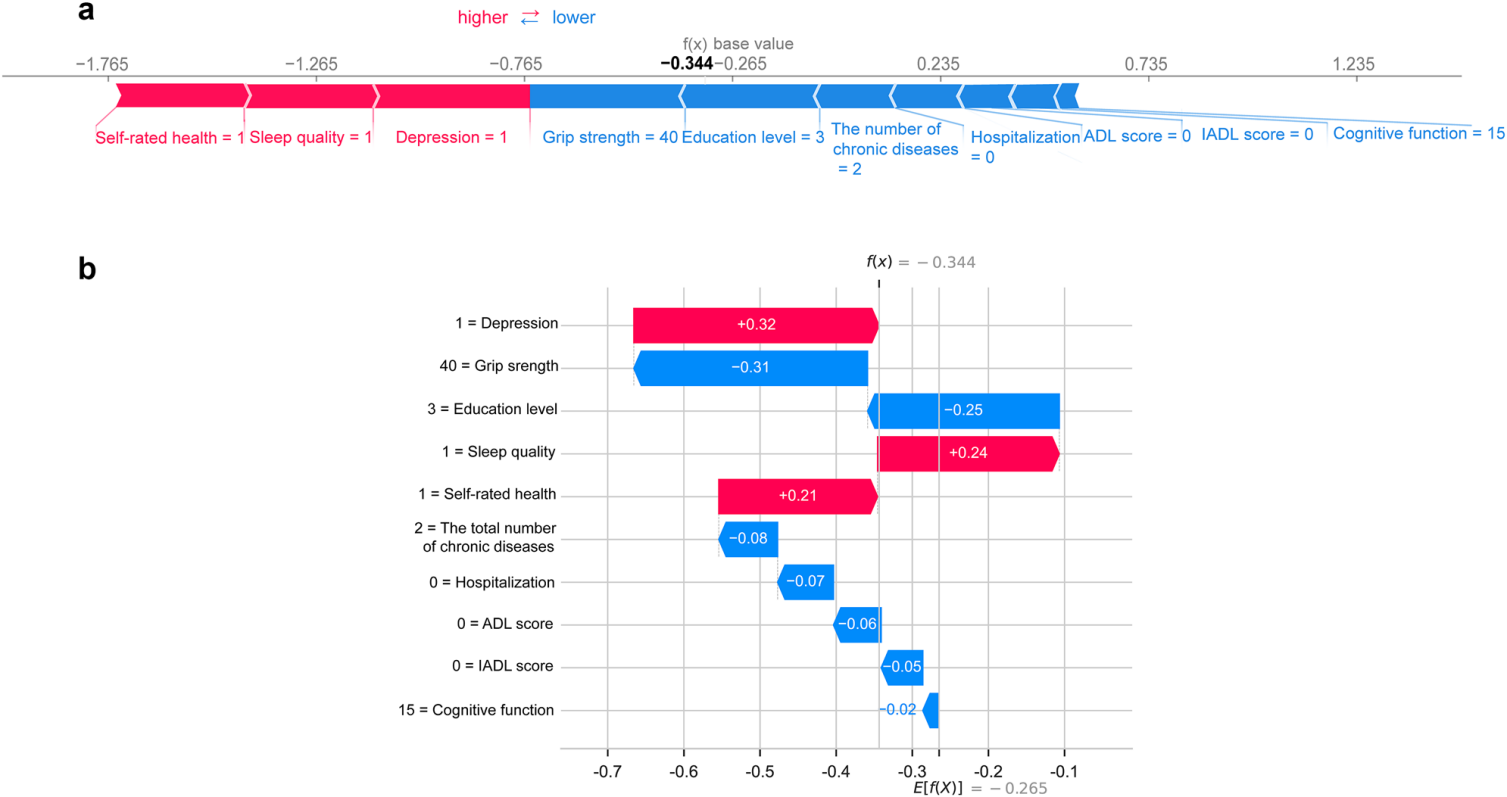
SHAP individual prediction visualization plots. (**a**) Force plot decomposing the prediction for a single participant. Red factors increase predicted risk; blue factors decrease risk. The base value (baseline prediction) is shown at the center. (**b**) Waterfall plot illustrating the step-by-step additive contribution of each feature to the final prediction for this representative case. SHAP, Shapley Additive exPlanations.

To illustrate individual-level predictions, a random participant was selected, and the LR model’s prediction was visualized (Fig. 7a). Red indicators represent factors that increase frailty risk, blue indicators represent protective factors, and f(x) values correspond to SHAP values. This participant’s predicted frailty risk was below the baseline, primarily influenced by their status regarding depression, grip strength, education level, sleep quality, and self-rated health. A detailed breakdown of how each feature contributed to this prediction is visually displayed in the waterfall plot (Fig. 7b).

### Correlation matrix of variables

To better understand the relationships among the final selected predictors, we performed a correlation analysis after feature selection for descriptive purposes. Supplementary Figure 1 shows correlations among the 10 key variables: depression was negatively correlated with self-rated health and sleep quality; IADL score was positively correlated with ADL score; and cognitive function was positively correlated with education level.

## Discussion

To our knowledge, this study addresses a gap in the literature by developing and validating an interpretable ML model to predict frailty risk specifically in individuals with GID. Frailty is considerably more prevalent among middle-aged and older adults with GID, and well-established epidemiological evidence identifies GID as an important risk factor for its development^8,9^. Potential mechanisms underlying this connection include age-related digestive decline, changes in gut microbiota composition, and the cumulative effects of GID and its treatments on physiology. These factors can lead to chronic inflammation, poor nutrient intake, malabsorption, malnutrition, sarcopenia, and ultimately, frailty^52^. This cumulative decline across multiple body systems, including the brain, endocrine system, immune system, and skeletal muscles, is key to the development of frailty^14^. Notably, patients with frailty in this demographic face significantly higher risks of major adverse health events, such as falls, fractures, loss of functional ability, reduced independence, increased reliance on long-term care, and premature death^53–55^. Therefore, early detection of high-risk cases is essential to implement preventive strategies and lessen these negative health outcomes, especially among individuals with GID.

To address this clinical need, we used the comprehensive CHARLS database to explore ML applications for frailty prediction, an understudied area in GID populations. We developed ten ML models (LR, MLP, NB, RF, GBC, LightGBM, SVM, AdaBoost, KNN, and DT) and systematically evaluated their performance to identify the optimal clinical tool. The LR model performed best on the testing set, with an AUC of 0.759 (95% CI: 0.711–0.806), accuracy of 0.685, sensitivity of 0.673, specificity of 0.691, precision of 0.503, and F1-score of 0.576. An AUC above 0.7 is generally considered clinically informative^56^, supporting the LR model as a valuable tool for assessing frailty risk in middle-aged and older adults with GID. In addition, the model demonstrated good calibration, as reflected by its calibration curves and Brier score, as well as favorable clinical utility, as indicated by DCA.

Using Boruta and LASSO regression, we selected 10 key predictors. Subsequent SHAP analysis identified influential risk and protective factors. Risk factors included depression, higher comorbidity burden, prior hospitalization, and impaired ADL/IADL function. Protective factors included greater grip strength, better self-rated health, higher education, improved sleep quality, and higher cognitive function. Notably, the top five predictors identified by SHAP were depression, grip strength, self-rated health, education level, and number of chronic diseases. These factors are discussed in detail below, along with their clinical implications.

Frailty development in GID patients is complex and multifactorial. Our analysis identified a significant link between depression and frailty in individuals with GID. This finding is consistent with earlier research using CHARLS data, which identified depressive symptoms as a predictor of frailty in adults aged 45 and above^57^. Further supported by studies from Brazil and Latin America^58,59^, depression appears to increase frailty risk in older adults, and this relationship may be stronger in middle-aged and older GID patients due to their higher prevalence of depressive disorders^60^. Common underlying mechanisms, such as chronic inflammation, oxidative stress, mitochondrial dysfunction, and dysregulation of the HPA axis, may help explain the interplay between depression and frailty^61–67^. We suggest that depressive symptoms, such as low mood, unintentional weight loss, and poor appetite, may lead to reduced physical activity, muscle weakness, and higher fall risk, thereby accelerating frailty.

We also found that grip strength was strongly associated with frailty, consistent with its role in the Fried frailty phenotype^20^. As an objective measure of muscle function and physiological reserve, grip strength provides a key indicator of overall physical capacity^68^. Its decline often signals reduced muscle mass and quality, resulting in impaired muscular performance and coordination that collectively contribute to the progression of frailty^69^. This is supported by the well-established pathophysiological features of frailty and sarcopenia, or loss of skeletal muscle mass^70,71^, underlining the physiological basis of our results.

Self-rated health was also closely tied to frailty. This measure integrates a person’s physical, mental, and social well-being with objective health status^72^. Previous studies support this relationship. For example, Huohvanainen et al. found that poor self-rated health in midlife predicted prefrailty, frailty, and mortality after 26 years^73^. Gijzel et al. reported that frail older adults had more variable self-rated health scores across multiple domains^74^. Similarly, Baddour et al. observed that better self-rated health was associated with a lower risk of frailty and preserved daily living skills^75^. Our results reinforce the value of self-rated health as a frailty indicator.

A lower educational level was also associated with higher prevalence of frailty, consistent with prior research. A 2017 Dutch study of 26,014 adults aged ≥55 years reported that lower education correlated with higher frailty scores^76^; an Italian study found higher education correlated with lower frailty scores^77^; and a Japanese national survey showed frailty was more common in older adults with lower education and household income^78^.

We also found that frailty was more common in GID patients with multiple chronic conditions or a recent hospitalization. Chronic diseases are known contributors to frailty^79^, and older adults experience complex interactions among comorbid conditions that may accelerate their progression^80^. Multimorbidity, defined as two or more chronic conditions, affects about 30% of people under 65 and 55–98% of those over 65^81,82^. One study confirmed that multimorbidity patterns are linked to physical frailty^22^, and others have established it as an independent risk factor^83^. A Chinese study also identified multimorbidity, having ≥3 chronic diseases, and recent hospitalization as independent frailty risk factors^84^. A Spanish ICU study similarly found that prior hospitalization increased frailty risk^85^. We suggest that polypharmacy related to multimorbidity raises the risk of drug interactions and reduces quality of life^86^, while hospitalization often leads to immobilization that worsens physical or psychological health^87^.

Poor sleep quality was also associated with frailty. Sleep problems are common in older adults; a meta-analysis in China found a 46% prevalence of sleep disorders^88^. Age-related sleep changes often disrupt sleep initiation and maintenance^89^. Previous studies consistently link poor sleep to frailty. A national survey in India found sleep disorders positively correlated with frailty^90^, and a prospective Chinese survey linked healthy sleep patterns to lower frailty risk^91^. Poor sleep is an established independent risk factor for frailty^92^. Potential mechanisms include sleep-related hormonal changes, such as reduced growth hormone, insulin-like growth factor, and testosterone, which increase muscle protein breakdown and promote geriatric syndromes like sarcopenia and frailty^93^.

Greater dependence in ADL and IADL was significantly correlated with elevated frailty risk among middle-aged and older adults with GID. ADL refers to self-care activities (e.g., bathing, dressing)^94^, while IADL refers to complex activities for independent living (e.g., cooking, shopping)^95^. This aligns with prior evidence. For instance, González-Bautista et al. reported that each additional impaired domain over five years raised frailty risk by 47%, with ADL and IADL impairments individually increasing risk by 23% and 27%, respectively^96^. Similarly, analysis of CHARLS data confirmed that both ADL and IADL disabilities significantly predicted frailty development over a two-year follow-up^97^.

Finally, we found that GID patients with poorer cognitive function had a higher risk of frailty. This aligns with studies linking frailty to subjective cognitive decline^98^. Shared mechanisms, such as chronic inflammation and oxidative stress, may drive both conditions^99^. Notably, GID is associated with disruption of gut microbiota^100^, and disturbances in the gut microbiota later in life can trigger inflammatory responses via the gut-brain axis, leading to inflammation in the central nervous system and impairing cognitive function^101^. In turn, cognitive decline can reduce self-management and treatment adherence in GID patients, worsening disease progression and increasing the risk of frailty.

The SHAP analysis identified several modifiable risk factors for frailty in this population, including depression, grip strength, self-rated health, education level, and chronic disease burden. These findings have direct clinical implications, consistent with previous studies that have used SHAP to translate machine learning outputs into actionable clinical insights^50,51^. First, screening for depressive symptoms and implementing mental health interventions may reduce frailty risk, given the strong association between depression and frailty. Second, promoting physical activity and muscle-strengthening exercises could help maintain grip strength and prevent frailty progression. Third, self-rated health serves as a simple yet informative indicator that can alert clinicians to patients at higher risk, prompting early evaluation. Fourth, addressing socioeconomic disparities, particularly low levels of education, through targeted support programs may help mitigate frailty risk among vulnerable populations. Finally, optimizing management of multimorbidity and reducing avoidable hospitalizations may lower frailty risk in patients with GID. By quantifying the contribution of each factor at the individual level, the SHAP-based model can assist clinicians in prioritizing interventions tailored to each patient’s risk profile, thereby facilitating personalized prevention strategies in line with established interpretable machine learning approaches^50,51^.

## Limitations and strengths

This study has several limitations that should be considered when interpreting the results. First, the use of self-reported measures for several key variables, including GID diagnosis and certain components of the frailty phenotype, may introduce recall bias or social desirability bias. Second, the lack of an external validation cohort is a major limitation. However, we used robust internal validation methods (train-test split with cross-validation); the model’s performance in independent populations remains unconfirmed. External validation across different cohorts is essential before clinical deployment. Third, as the data were collected solely from a Chinese population, the applicability of our model to other ethnic or regional groups remains unclear. Fourth, the prediction horizon was fixed at approximately two years based on the CHARLS data structure; therefore, the model’s performance for longer or shorter prediction windows remains unknown and should be evaluated in future studies. Future studies including more diverse cohorts are needed to improve the external validity of the findings.

Despite these limitations, our study has several notable strengths. The analysis was conducted using the CHARLS database, a high-quality, nationally representative cohort that provides reliable and comprehensive health data. We employed a rigorous feature selection process combining the Boruta algorithm and LASSO regression, which helped prevent overfitting while maintaining clinical interpretability by identifying only 10 key predictors, all of which are easily assessed in routine clinical practice. The final LR model demonstrated moderate discriminative ability. Moreover, applying the SHAP framework significantly enhanced model interpretability, providing clinicians with transparent and actionable insights into individual prediction mechanisms.

Despite these strengths, a key consideration should be noted before clinical implementation. The LR model demonstrated acceptable discriminative ability (AUC = 0.759) and good calibration, indicating its potential as a screening tool for frailty risk in middle-aged and older adults with GID. However, it is not meant to replace clinical judgment but to complement it by highlighting modifiable risk factors (e.g., depression, grip strength, chronic disease burden) that may guide targeted interventions. Acknowledging these limitations, the model should be considered a decision-support tool requiring prospective evaluation in real-world clinical settings before widespread adoption.

Looking forward, several directions emerge for further research. First, to facilitate clinical adoption, we plan to convert the current LR model into a user-friendly tool, such as a web-based risk calculator or a simplified point-score system. This would allow clinicians to estimate frailty risk in real time without specialized software, supporting its integration into routine clinical practice. Second, future studies could evaluate the model’s performance across different prediction horizons (e.g., 1-year, 5-year) to better understand its temporal generalizability. Third, expanding the study to include larger, more demographically diverse populations spanning different geographic regions, socioeconomic backgrounds, and spectra of gastrointestinal disease severity would help verify the model’s robustness and general applicability. Fourth, integrating multimodal data sources, such as electronic health records, medical imaging, and real-time data from wearable devices, could substantially improve predictive accuracy. Advanced modeling techniques, including convolutional and recurrent neural networks, may be well-suited to leverage such complex datasets, although these approaches are beyond the scope of the current work^102–104^. Finally, randomized controlled trials are warranted to evaluate the real-world clinical utility of the proposed model, specifically, whether its implementation leads to meaningful improvements in patient outcomes, such as reduced frailty incidence or enhanced treatment adherence among middle-aged and older adults with GID.

## Conclusion

We developed and compared ten ML algorithms to predict frailty in Chinese middle-aged and older adults with GID, using clinically relevant data from the CHARLS database. The LR model performed best, showing good discriminative ability and stability. The SHAP-based interpretable model clarified key frailty risk factors, providing a promising framework for future development of a practical clinical tool. Future research should evaluate the LR model’s generalizability across healthcare settings and explore its translation into user-friendly formats to support its integration into routine clinical practice.

## Supplementary Information

Supplementary Information 1.

Supplementary Information 2.

Supplementary Information 3.

## Data Availability

The dataset supporting this study’s findings is publicly accessible and can be accessed directly from the official CHARLS portal at https://charls.charlsdata.com/.

## Code availability

The custom code generated for data processing, feature selection, model development, evaluation, and SHAP analysis in this study is publicly available at https://github.com/qiaoye1991/frailty-prediction-charls.
