# Supplementary material for: An interpretable machine learning model predicts frailty risk in middle-aged and older adults with gastrointestinal disease: a longitudinal study
Source: Sci Rep. 2026 Apr 28;16:19654. doi: 10.1038/s41598-026-50348-x (PMC13315625; doi:10.1038/s41598-026-50348-x)
Supplement: Supplementary file 1 — Supplementary Information 1. [file 41598_2026_50348_MOESM1_ESM.docx]

**Supplementary Figure 1.** Correlation matrix of the 10 key predictors selected by Boruta and LASSO. This analysis was performed after feature selection for descriptive purposes and did not influence predictor selection. Blue indicates positive correlations, red indicates negative correlations, and color intensity reflects correlation strength.
