# Supplementary figures and images for: An interpretable machine learning model predicts frailty risk in middle-aged and older adults with gastrointestinal disease: a longitudinal study
Source: Sci Rep. 2026 Apr 28;16:19654. doi: 10.1038/s41598-026-50348-x (PMC13315625; doi:10.1038/s41598-026-50348-x)

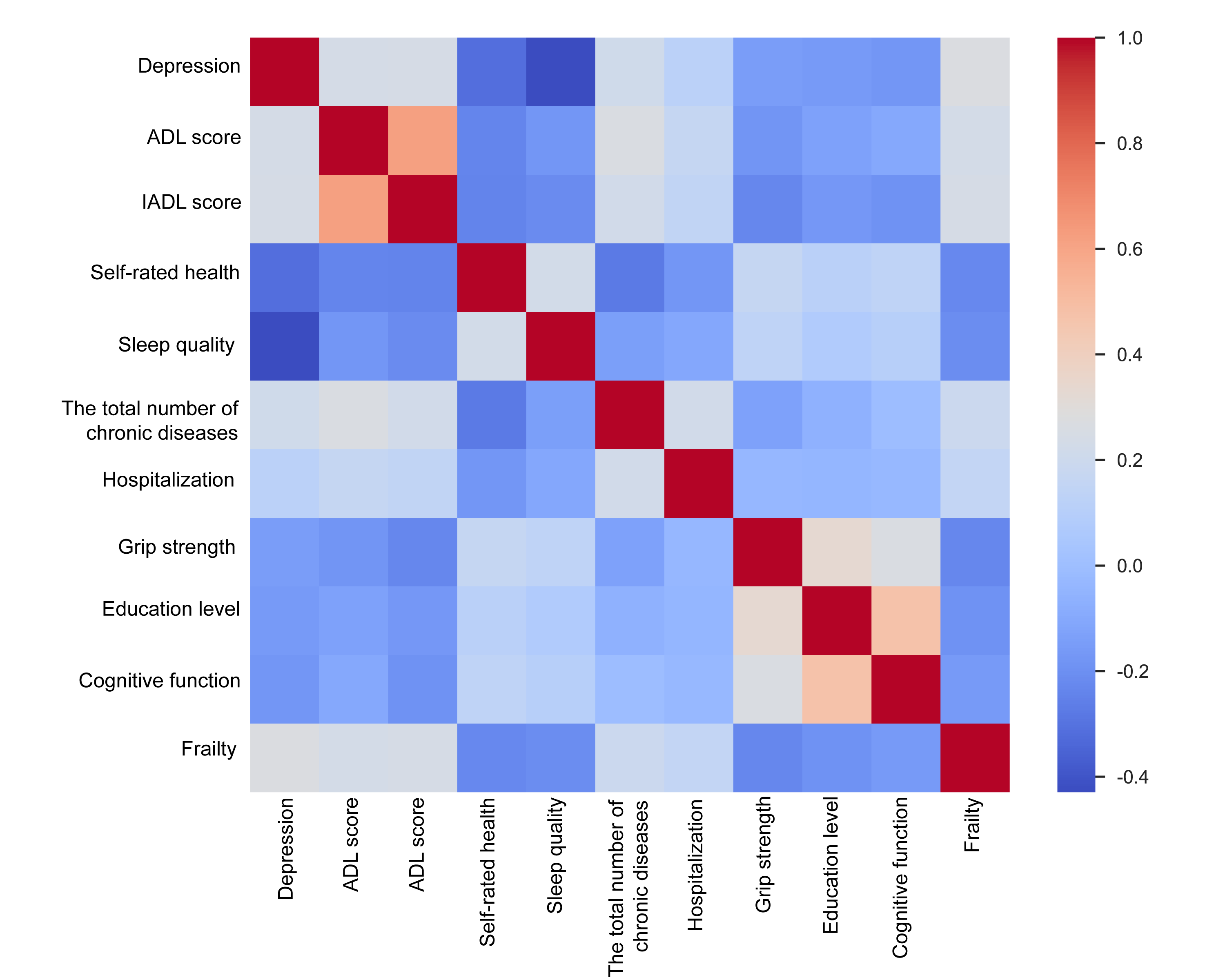

Supplement: Supplementary file 2 — Supplementary Information 2. [file 41598_2026_50348_MOESM2_ESM.tif]
