# Supplementary material for: An interpretable machine learning model predicts frailty risk in middle-aged and older adults with gastrointestinal disease: a longitudinal study
Source: Sci Rep. 2026 Apr 28;16:19654. doi: 10.1038/s41598-026-50348-x (PMC13315625; doi:10.1038/s41598-026-50348-x)
Supplement: Supplementary file 3 — Supplementary Information 3. [file 41598_2026_50348_MOESM3_ESM.docx]

**Supplementary Table 1. Optimal hyperparameters of the ten models.**

| **Model** | **Key Hyperparameters** |
| --- | --- |
| **LR** | C: 1.0  penalty: ‘l2’  solver: ‘lbfgs’  max_iter: 1000 |
| **MLP** | activation: ‘logistic’  alpha: 2.7199e-07  hidden_layer_sizes: [76, 67, 10]  learning_rate: ‘adaptive’ |
| **NB** | var_smoothing: 0.00185 |
| **SVM** | alpha: 0.000135  learning_rate: ‘adaptive’  loss: ‘hinge’  penalty: ‘elasticnet’  l1_ratio: 0.5195  eta0: 0.002116 |
| **AdaBoost** | algorithm: ‘SAMME.R’  learning_rate: 1.0  n_estimators: 50 |
| **KNN** | n_neighbors: 36  metric: ‘euclidean’  weights: ‘uniform’ |
| **RF** | n_estimators: 185  max_depth: 6  max_features: 0.5825  min_samples_leaf: 5  min_samples_split: 5 |
| **GBC** | learning_rate: 0.0133  max_depth: 2  n_estimators: 192  subsample: 0.2129  max_features: 0.8173  min_samples_leaf: 3  min_samples_split: 7 |
| **LightGBM** | learning_rate: 0.0143  n_estimators: 137  num_leaves: 89  feature_fraction: 0.5095  bagging_fraction: 0.5053  bagging_freq: 4  min_child_samples: 54  min_split_gain: 0.0597 |
| **DT** | max_depth: 6  max_features: 0.6489  min_samples_leaf: 6  min_samples_split: 4  min_impurity_decrease: 1.5918e-05 |

LR, Logistic Regression; MLP, Multilayer Perceptron; NB, Naive Bayes; SVM, Support Vector Machine; Ada Boost, Adaptive Boosting; KNN, k-Nearest Neighbors; RF, Random Forest; GBC, Gradient Boosting Classifier; LightGBM, Light Gradient Boosting Machine; DT, Decision Tree.
